# Supplementary material for: Chromothripsis during telomere crisis is independent of NHEJ, and consistent with a replicative origin
Source: Genome Res. 2019 May;29(5):737–49. doi: 10.1101/gr.240705.118 (PMC6499312; doi:10.1101/gr.240705.118)
Supplement: Supplemental Material [file supp_gr.240705.118_Supplemental_file_1.zip › contigs/annotated_contigs/DB37/contig.3.DB37_length_579_mean_cov_4.6597582038.docx]

**DB37_length_579_mean_cov_4.6597582038**

```
CCTTGCGATAGTATTGCTGCTATAAAGACACATGCACATGTATGTTTATTGTGGAATTATTCACAATAGCAAAGACTTGGAACCAACCC
 >chr9:22531826-22531990 + E=6e-84 p=0e+00
AAATGTCCAACAAT|GATAGACTGGATTAAGAAAATGTGGCACATATACACCATGGAATACTATGCAGCCATAAAA|TAGAATGAGATC
               >chr16:72356105-72356393 - E=1e-146 p=3e-02
ATGGTCTTTGCAGCAACGTGTATAGAGCTAGAGGCCAGTATCCTAAGTGAACTAATGCAGGAACAGAAAACCAAATACTGCATGTTCTC

ACTTGTAAGTGGAAGCTAAACATTGAGTTCACATGGACACAGAGTAGGGAACAACAGACACTGGGATGTACTTGAGAGAGTGGGAGGAG

GATGAGGATTAAAAAACCATCTGCCACATACTATG|CT|AATACAGTGAATTGGTACCAAGGTAGTGAGGCACTGCTGTAAAGATACCT
                                    >chr16:72354579-72354769 - E=1e-102
GAAAATGTGGAGTGACCTTGGGACTGGGTAATAGGCAGAGGTTGGAACAGTTTGGAGGGCTCTGAAGAAGACAGGAAAATGTGGGAAAG

TTTGGAGTTTCCTAGAGACTTGGAGGGCTCAGAAGACAGAAAGATTTGG
```
